# Unilateral Neurological Deficit Due to Spinal Epidural Hematoma Following Midline-Sparing Spine Surgery: A Case Report

**DOI:** 10.7759/cureus.50788

**Published:** 2023-12-19

**Authors:** Gururaj Sangondimath, Jomin George, Fazal Rehman T, Amlan Singh, Mayukh Guha

**Affiliations:** 1 Spine Surgery, Indian Spinal Injuries Center, Delhi, IND

**Keywords:** epidural hematoma in tlif, bilateral drain application in midline sparing spine, spinal epidural hematoma (seh), postoperative spinal epidural hematoma, unilateral epidural hematoma

## Abstract

Symptomatic spinal epidural hematoma (SEH) is a rare but well-documented complication in spine surgery, often associated with risk factors such as abnormal coagulation parameters, low platelets, excessive epidural bleeding, and inadequate hemostasis. While bilateral SEH is frequently described in the literature, unilateral SEH following spine surgery is seldom reported. We present a unique case of a unilateral neurological deficit resulting from an SEH following midline-sparing spine surgery due to unilateral drain placement in an 80-year-old male patient without comorbidities and normal coagulation parameters. Subsequent evacuation of the hematoma was done leading to gradual recovery of neurology. This emphasizes the importance of bilateral drain placement in such midline-sparing spine surgeries. This report underscores the significance of early SEH diagnosis and intervention, providing valuable insights into preventive measures and the need for a high index of suspicion in managing this potentially debilitating complication.

## Introduction

Spinal epidural hematomas (SEHs) are space-occupying lesions characterized by the settling of blood between the dura mater and bone or ligament components, which places pressure on the spinal cord causing the progressive neurological deficit. It is a rare but well-documented complication in spine surgery. While computed tomography scans and magnetic resonance images have found asymptomatic epidural hematomas in 33-100% of patients following lumbar decompression surgery [1], the incidence rate of SEH is only in 0.1-0.2% of patients [2]. The major risk factors for the condition include abnormal coagulation parameters, low platelets, excessive epidural bleeding, and inadequate hemostasis at the time of wound closure. Though there are many studies in literature describing bilateral epidural hematoma, unilateral epidural hematoma following spine surgery is seldom described. In this case report, we present a case of an 80-year-old male patient without any comorbidities, with normal coagulation and blood parameters, who developed the unilateral epidural hematoma due to a contralateral placement of a drain in a midline-sparing case of spine surgery.

## Case presentation

**Figure 1:**
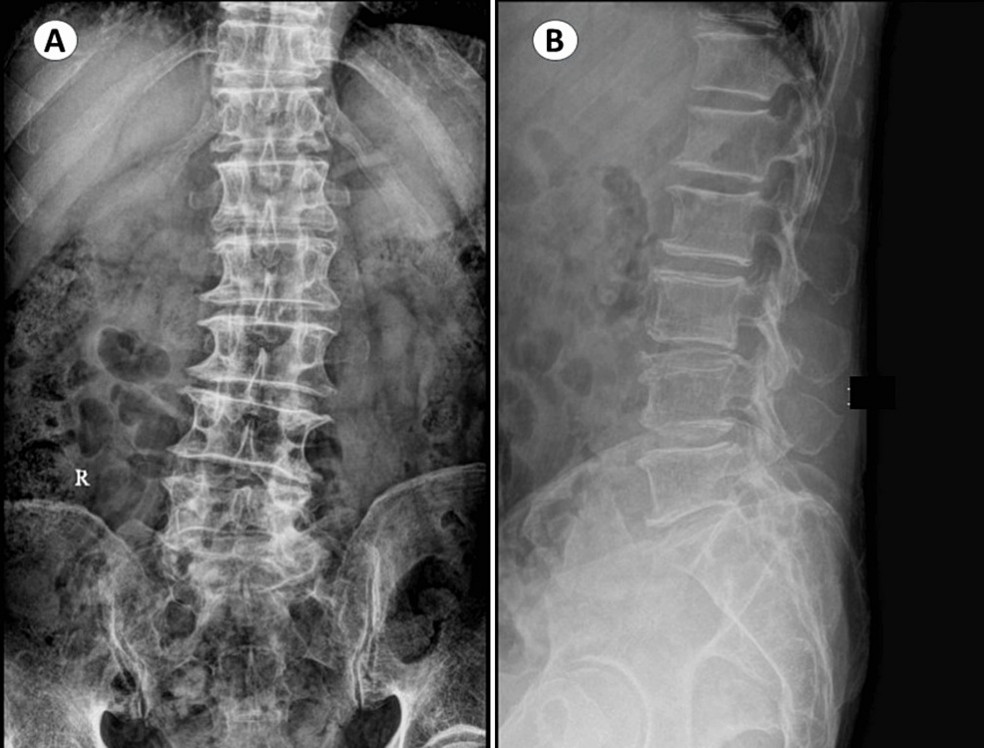
Preoperative X-ray lumbosacral spine anteroposterior view (A) and lateral view (B) showing asymmetric disc collapse and degenerative scoliosis involving the L3-L4 and L4-L5 segments

**Figure 2:**
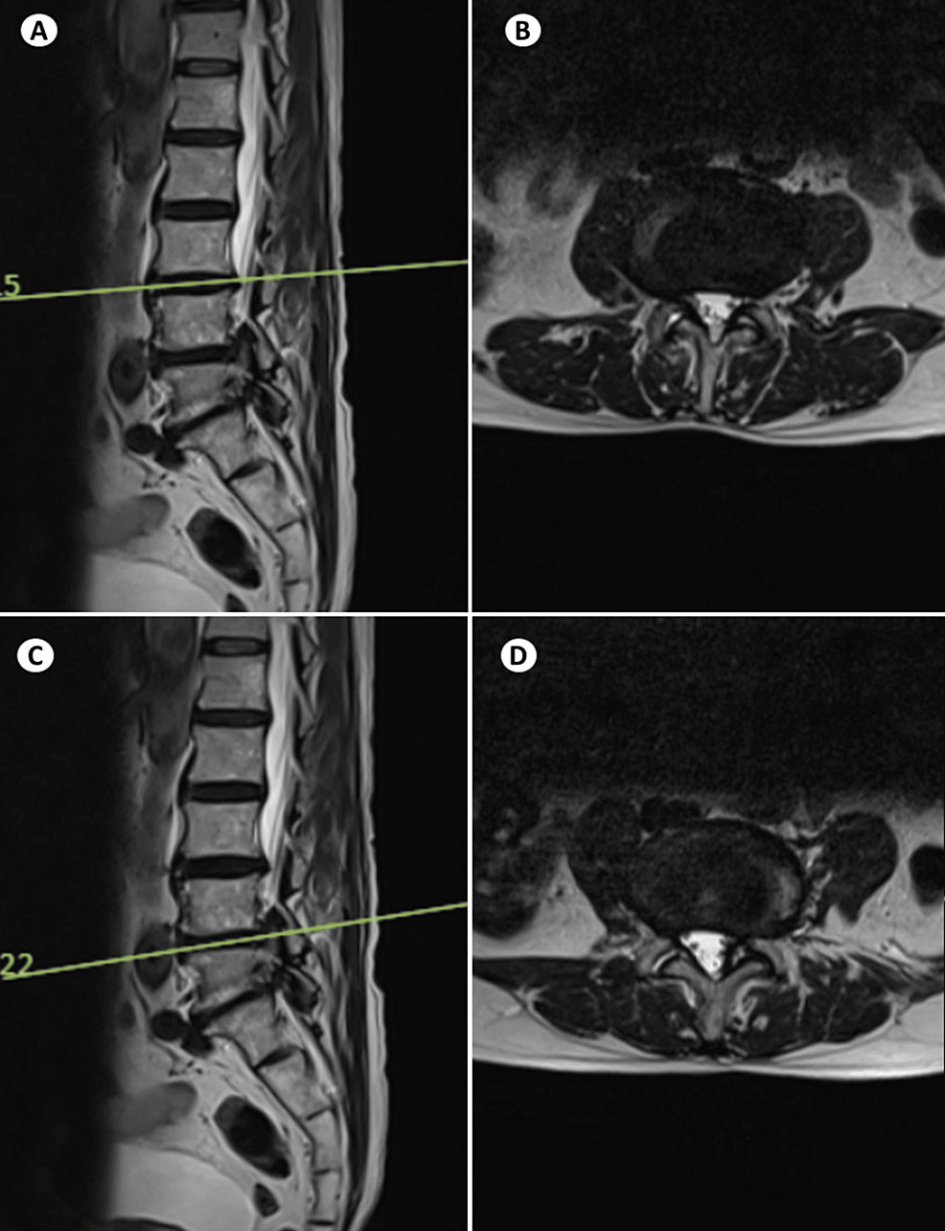
Preoperative MRI lumbosacral spine T2 view right para sagittal (A) and axial (B) of the L3-L4 disc space and right para sagittal (C) and axial (D) of the L4-L5 showing severe foraminal stenosis and facet effusion

An 80-year-old male patient without any comorbidities presented with complaints of severe back pain and right-sided buttock pain. Back pain was present for a period of seven years and leg pain for a period of six months. However, the pain continued to persist despite the conservative management. The patient reported to our spine clinic with complaints of aggravation of leg pain. He was evaluated and found to have asymmetric disc collapse and degenerative scoliosis involving L3-L4 and L4-L5 segments (Figure 1). His neurology was normal except for right extensor hallucis longus being grade 4. Figure 2 shows the preoperative magnetic resonance imaging with sagittal and axial views of L3-L4 and L4-L5 disc spaces showing severe foraminal stenosis and facet joint effusion.

Since the patient did not have relief despite six months of conservative management with physiotherapy, analgesics, nerve blocks, and spinal strengthening exercises, a decision was made to undergo surgical management. He was evaluated by a multidisciplinary team of orthopedic spine surgeons, physicians, anesthesiologists, and cardiologists and was taken up for surgery after confirming coronavirus disease 2019 (COVID-19) negative status. His preoperative blood coagulation parameters were all normal. Deformity correction by transforaminal lumbar interbody fusion L3-L4 from the right side and L4-L5 from the left side by midline-sparing approach was done. A closed wound suction surgical drain was applied on the left side of the midline and was maintained under negative suction pressure. The immediate post-procedure period was uneventful. He was kept on intermittent pneumatic compression devices for mechanical prophylaxis for DVT. No pharmacological DVT prophylaxis was given as the patient was ambulatory in the preop period.

**Figure 3:**
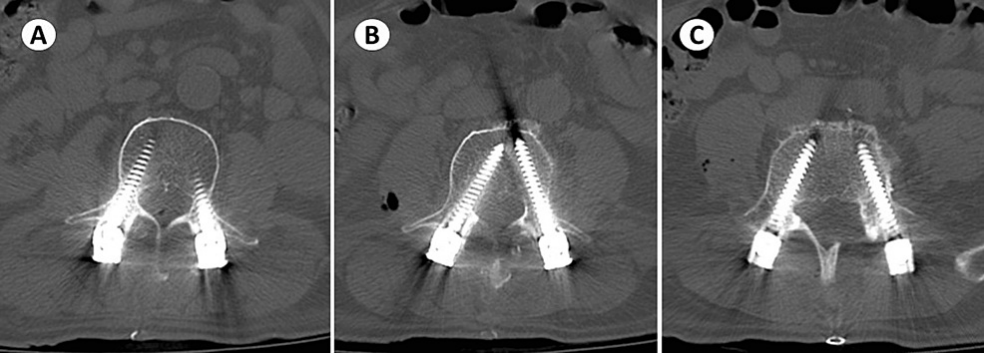
Postoperative CT scan axial views of L3 screws (A), L4 screws (B), and L5 screws (C) showing acceptable trajectories, which out any cortical breach

**Figure 4:**
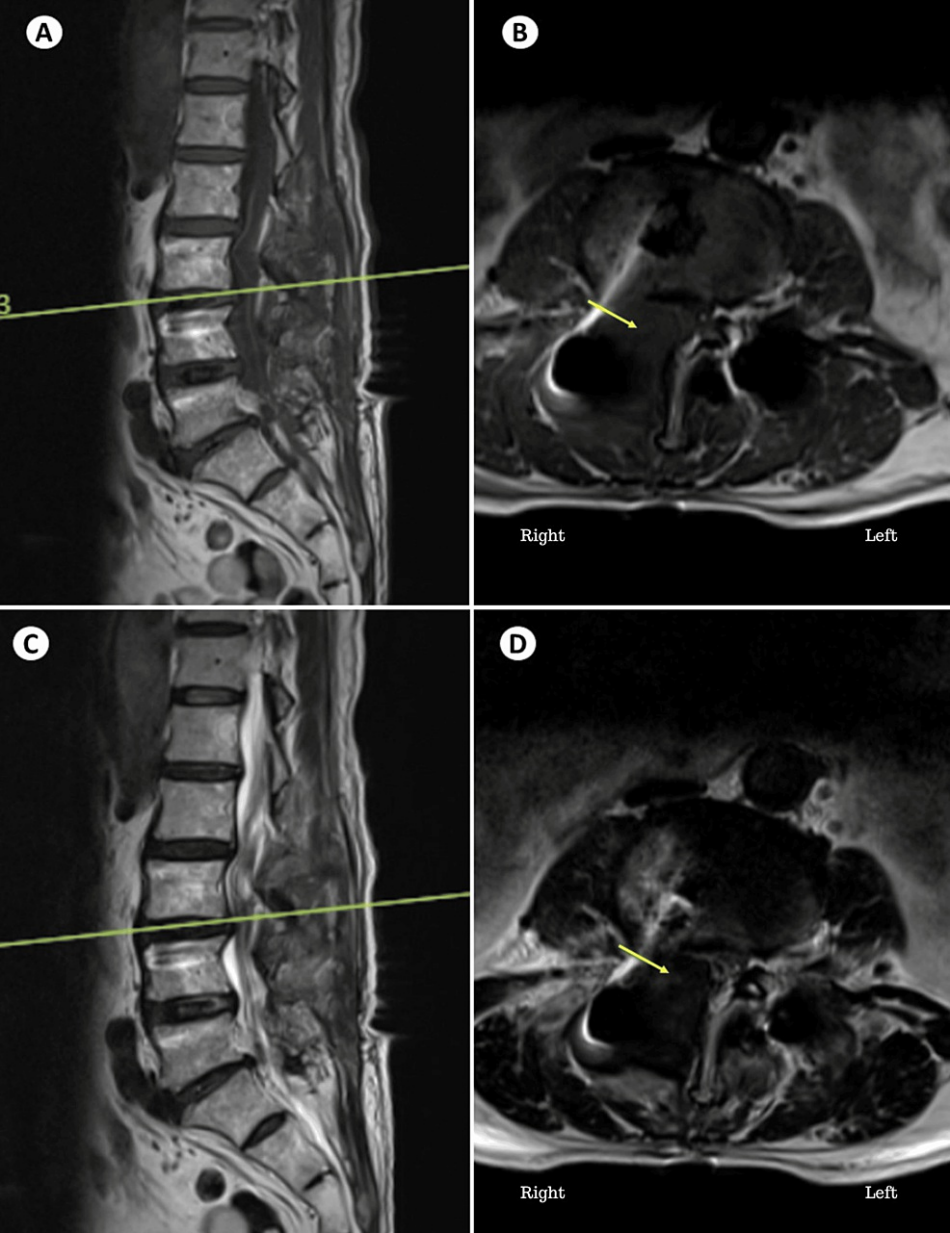
Postoperative MRI lumbosacral spine T1 view sagittal (A) and axial (B) showing an iso-intense lesion (yellow arrow) over the right L3-L4 facetectomy defect area and T2 view sagittal (A) and axial (B) showing a heterogenous hyperintense T2 lesion (yellow arrow) over the right L3-L4 facetectomy defect area leading to suspicion of an epidural hematoma

The patient was reassessed the next day morning. He complained of numbness and pain in the right anterior thigh and difficulty in extending the right knee. There was no soakage of dressing, and the drain output was 250 ml. He was evaluated and found to have grade 2 power in right knee extensors and ankle dorsiflexors were grade 4 and right extensor hallucis longus was grade 4. The patient was given intravenous methylprednisolone given neurological deficits but there was no improvement of symptoms. He was re-investigated by X-ray lumbosacral spine, CT lumbosacral spine, and magnetic resonance imaging of the lumbosacral spine to look for possible screw breach, implant failure, and for the presence of persistent disc fragments or any other source of compression. The postoperative CT scan (Figure 3) was unremarkable with all screws in acceptable trajectories. Magnetic resonance imaging of the lumbosacral spine showed iso intensity in T1 and heterogenous hyperintense T2 lesion over the facetectomy defect on the right side over the L3-L4 region (Figure 4), leading to suspicion of an epidural hematoma.

**Figure 5:**
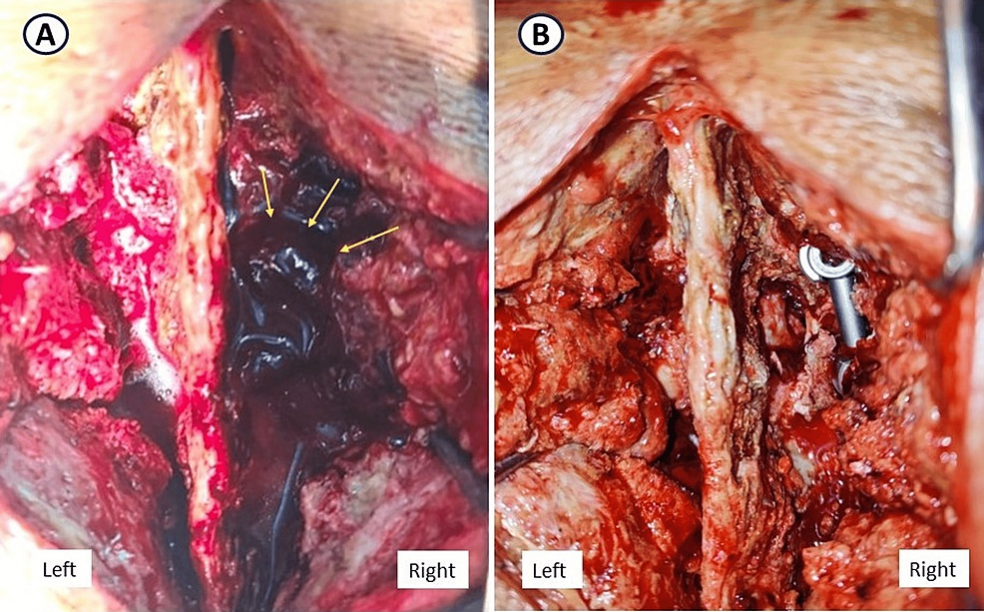
Intraoperative image (A) showing a large hematoma (yellow arrows) present over the right L3-L4 facetectomy defect compressing the exiting and traversing roots. Intraoperative image (B) showing visible dura and root after hematoma evacuation.

As there was continued worsening of symptoms due to pain, re-exploration of the surgical wound was done. A large hematoma was present over the right L3-L4 facetectomy defect compressing the exiting and traversing roots (Figure 5). The left side lamino-facetal region was found to be normal without any clots, as the drain was placed on the left side. The left-sided drain was not able to drain the blood collected on the right side, as a midline preserving surgery was done, as such hematoma got collected on the right side leading to a right-sided postoperative neurological deficit.

Hematoma removal was done, and nerve roots were freed of any compression (Figure 5). Postoperatively, the patient recovered over a period of two weeks to grade 4 power of knee extensors and the rest of the neurology remained the same. His preoperative symptoms of back pain and buttock pain settled. Post-op follow-up at six months showed complete recovery of neurology and sensory symptoms. Following the incident, the author has started using two drain tubes, on both sides of the spinous process, in surgeries where midline spinous process preservation was done.

## Discussion

Symptomatic spinal epidural hematoma is one of the well-documented causes of postoperative neurological complications, including persistent pain, numbness, and neurological deficit. It is defined as a spinal epidural hematoma that requires a second surgery for evacuation [3]. The risk factors for the condition include preoperative coagulopathy (abnormal coagulation parameters, low platelets) and multilevel surgical procedures [4]. This condition should be suspected in a patient who develops a neurological deficit in the immediate postoperative period [4]. Usually, the condition presents with bilateral symptoms; however, cases of patients with unilateral neurological deficits are seldom reported [5,6].

SEH is broadly divided into two types, early SEH, which develops within 24 hours, and delayed-onset SEH), which develops after three days of surgery. There have also been reports of SEH developing even two weeks after surgery [7]. The risk factors for early SEH include multilevel procedures involving more than one intervertebral disc, postoperative systolic BP, and an abnormal coagulation profile, whereas the risk factors for late SEH include previous spinal surgeries and postoperative systolic BP [8]. The suspected mechanisms by which hypertension leads to SEH include hardening of the vessels and increasing the viscosity of blood, which causes blood clots [8].

SEH may develop after a lumbar puncture or epidural analgesia. Anticoagulation, thrombolysis, thrombocytopenia, neoplasms, blood dyscrasias, coagulopathies, and vascular malformations are additional factors associated with this condition [9]. Spontaneous spinal epidural hematoma is defined as blood within the epidural space without any obvious traumatic or iatrogenic reasons. Arteriovenous malformations and coagulopathies may be potential associations [9]. Iatrogenic causes are more common and in our study, we describe a case of iatrogenic spinal epidural hematoma causing unilateral neurological deficit. In recent times, COVID-19 [10] and its vaccines [11] have also been proposed as a potential cause of SEH.

In a recent meta-analysis by Chen et al., it has been identified that the incidence of SEH is higher when surgery is done for deformity and tumor surgeries rather than for degeneration [3]. They also found that the incidence of SEH is five times higher in minimally invasive spine surgeries compared to open surgeries [12,13]. The probable reason is that the bleeding is masked by continuous saline irrigation, which masks the epidural venous bleeding that collapses the veins during the procedure [12,14]. However, gender and preoperative anticoagulation were not identified as a significant risk factor for SEH [3,15]. Though in the literature, enoxaparin and rivaroxaban are used in postoperative anticoagulation prophylaxis, a considerable number of clinicians continue to be concerned that early initiation of anti-coagulation may increase the risk of postoperative rebleeding; consequently, it is advised that the timing, agent, and dosage be individualized [16]. Contrary to the same, in a systematic review by Chen et al., it has been found that there is no role of preoperative chemoprophylaxis in the development of post-operative spinal epidural hematoma in open spine surgeries [3].

In SEH, rapid identification is essential in order to avert substantial morbidity and mortality, and risk factors include age greater than 60 years, weekly alcohol consumption exceeding 10 drinks, preoperative use of nonsteroidal anti-inflammatory drugs, a prior history of spine surgery, and multiple-level spine surgery [17]. Similar to SEH, one of the rare situations when the timing of the intervention affects the result is cauda equina syndrome, which is one of the emergency scenarios in spine surgery [18]. Short duration of symptoms and rapid intervention are correlated with better clinical outcomes [19,20] and complete neurological recovery [21]. According to Yamada et al., hematomas that were evacuated within 24 hours of commencement of symptoms had noticeably better results than those that were operated on beyond 24 hours [22]. Few studies have suggested that the lack of a drainage tube or insufficient postoperative drainage can be risk factors for SEH [6,19,23]. However, another study by Kanayama et al. showed that the use of a suction drain does not influence the development of postoperative hematoma [24]. Optimizing the patient before surgery in terms of coagulation parameters and meticulous hemostasis before closure are strategies that can reduce the chance of occurrence of this complication [25].

In our case, as it was a midline-sparing surgery, unilateral drain application was not able to drain the hematoma formation on the contralateral side. This emphasizes the importance of bilateral drain placement in such midline-sparing spine surgeries. This report underscores the significance of early SEH diagnosis and intervention, providing valuable insights into preventive measures and the need for a high index of suspicion in managing this potentially debilitating complication.

## Conclusions

Symptomatic spinal epidural hematoma is a rare but preventable condition leading to complications like neurological deficit, pain, and other sensory symptoms. Timely identification, by maintaining a high index of suspicion and prompt intervention when SEH has been diagnosed, can prevent catastrophic complications. Optimizing the patient before surgery in terms of blood counts and coagulation parameters, meticulous hemostasis before closure by properly cauterizing muscular bleeding points and epidural veins, applying bone wax to the bleeding edges of bone, controlling the perioperative blood pressure, and proper drain placement with special emphasis on bilateral drain placement in cases of midline-sparing surgeries are the important measures a spine surgeon can take to prevent the occurrence of SEH.
